# Renal effects of a sodium‐glucose cotransporter 2 inhibitor, tofogliflozin, in relation to sodium intake and glycaemic status

**DOI:** 10.1111/dom.13731

**Published:** 2019-05-06

**Authors:** Kiyohide Nunoi, Yuichi Sato, Kohei Kaku, Akihiro Yoshida, Hideki Suganami

**Affiliations:** ^1^ Division of Endocrinology and Metabolism St. Mary's Hospital Fukuoka Japan; ^2^ Department of General Internal Medicine Kawasaki Medical School Okayama Japan; ^3^ Medical Information and Product Advancement Department Kowa Pharmaceutical Company, Ltd. Tokyo Japan; ^4^ Clinical Data Science Department Kowa Company, Ltd. Tokyo Japan

**Keywords:** daily salt intake, glomerular filtration rate, sodium‐glucose cotransporter 2 inhibitor, tofogliflozin

## Abstract

**Aims:**

Little is known about whether sodium intake is associated with the clinical effects of SGLT2 inhibitors (SGLT2is); however, SGLT2is may increase urinary sodium excretion. Thus, we investigated the impact of daily sodium intake on the estimated glomerular filtration rate (eGFR) via an SGLT2i, tofogliflozin (TOFO), in patients with type 2 diabetes (T2D).

**Methods:**

Individual‐level data on 775 T2D patients in TOFO Phase 3 trials were analysed. Adjusted changes in variables during 52 weeks of TOFO therapy were compared according to basal daily salt intake (DSI), which was measured based on estimated daily urinary sodium excretion using the Tanaka formula. Multivariable analysis was used to investigate the impact of basal DSI on changes in eGFR at Weeks 4 and 52.

**Results:**

Sixty‐six percent of participants were men; mean age, HbA1c, body mass index, eGFR_MDRD_ and median DSI were 58.5 years, 8.0%, 25.6 kg/m^2^, 83.9 mL/min/1.73 m^2^ and 9.3 g/d, respectively. In all participants, eGFR_MDRD_ sharply dipped during Week 4, and gradually increased by Week 52, showing a significant increase overall from baseline to Week 52. Multivariable analysis showed that basal DSI and HbA1c levels were independently correlated with eGFR_MDRD_ changes at Weeks 4 and 52. Additionally, lower baseline HbA1c and DSI levels were significantly correlated with a greater increase in eGFR_MDRD_ at Week 52.

**Conclusions:**

Dietary salt intake, in addition to glycaemic control, correlates with changed eGFR_MDRD_ via TOFO. Thus, an appropriate dietary approach to therapy should be considered before treatment of T2D patients with an SGLT2i.

## 1 INTRODUCTION

Type 2 diabetes (T2D) is the leading cause of renal and cardiovascular disease in the world.^1^ Hyperglycaemia as the result of diabetes is thought to be exaggerated by hyper‐reabsorption of renal glucose in the proximal tubule. However, the etiology of the increased glycosuria threshold in T2D is still unclear. One of the mechanisms of hyper‐reabsorption of glucose is an increased expression of sodium/glucose cotransporter‐2 (SGLT2) in patients with T2D and diabetic nephropathy.^2^, ^3^ Increased SGLT2 induces increased proximal tubular reabsorption of, not only glucose, but also sodium, both of which are SGLT2 substrates. This reduces sodium chloride and fluid delivery from the proximal tubule to the downstream macula densa, causing glomerular hyperfiltration via impaired tubuloglomerular feedback (TGF).^4^ Finally, glomerular hyperfiltration exaggerates the work of sodium transport and oxygen consumption in the kidney, particularly in the proximal tubules,^5^ leading to subsequent kidney damage. Therefore, diabetes‐induced glomerular hyperfiltration is one of the major risk factors for the subsequent development of diabetic kidney disease.^6^

High intake of dietary sodium was associated with an elevated incidence of cardiovascular disease in patients with T2D,^7^ while urinary sodium excretion was nonlinearly associated with all‐cause mortality and the cumulative incidence of ESRD.^8^ Lower urinary sodium excretion was reported to be associated with increased all‐cause and cardiovascular mortality in T2D patients.^9^ Therefore, sodium intake may be associated with risks of cardiovascular and kidney disease. Also, dietary sodium intake was reported to influence renal haemodynamics**.** 10 However**,** little is known about the mechanism of the effects of sodium intake on renal haemodynamics.

The SGLT2 reabsorbs, not only filtered glucose, but also sodium. In diabetes, increased expression and activity of SGLT2, and fully activated SGLT1, account for almost 50 g of sodium, which may represent over 10% of the filtered sodium load, may be reabsorbed via SGLT‐dependent pathways.^11^ Although post meal urinary sodium excretion, in addition to urinary glucose excretion, was increased from baseline, both acutely and chronically, by administration of an SGLT2 inhibitor (SGLT2i),^12^ little is known about the association of sodium intake with the clinical effects of SGLT2is. Fundamental experiments indicated that genetic and pharmacological inhibition of SGLT2 attenuated primary proximal tubule hyper‐reabsorption of sodium and glucose in diabetic models and, thereby, lowered glomerular hyperfiltration via TGF.^13^, ^14^ Additionally, lowering of the glomerular filtration rate (GFR) via the SGLT2i, empagliflozin, was reported in patients with type 1 diabetes.^1^ Recently, striking reductions in the relative risk of, not only cardiovascular, but also renal, outcomes with use of SGLT2is in patients with T2D were observed in the EMPA‐REG OUTCOME trial, the CANVAS Program and the DECLARE–TIMI 58 study.^16^, ^17^, ^18^, ^19^ However, the renal effects, particularly those on the estimated glomerular filtration rate (eGFR) at different levels of baseline sodium intake estimated from urinary sodium excretion, have not been investigated. We therefore investigated the impact of basal salt intake on changes in the eGFR in patients with T2D using an SGLT2i, tofogliflozin (TOFO), focusing on early and chronic effects, as well as effects two weeks after the termination of treatment.

## 2 RESEARCH DESIGN AND METHODS

A pooled analysis was conducted on two Phase 3 studies (Table S1) of administration of TOFO to patients with T2D. Various doses of TOFO, either as monotherapy or as an adjuvant antidiabetic agent, were compared. The CSG004JP study (TOFO, 20 and 40 mg monotherapy) and the CSG005JP study (TOFO, 20 and 40 mg as add‐on to other oral antidiabetic agents) were both 52‐week, randomized, controlled, open‐label, Phase 3 studies.^20^ Individual‐level data from the 52‐week core treatment and 2‐week termination of treatment periods of each study were used for analysis. Each included study was conducted in accordance with the Declaration of Helsinki and Good Clinical Practice. Protocols were reviewed and approved by the institutional review boards of each participating center. All patients provided written informed consent prior to enrollment.

The following laboratory variables were measured at baseline: HbA1c, fasting plasma glucose (FPG), sodium, potassium, uric acid, brain natriuretic peptide (BNP), urine creatinine, urine sodium, urine potassium, urine albumin‐to‐creatinine ratio (ACR), creatinine and cystatin C. The eGFR of creatinine (eGFR_MDRD_) was estimated using the Modification of the Diet in Renal Disease (MDRD) formula for the Japanese population^21^, ^22^ and the eGFR_CKD‐EPI_ was estimated using the Chronic Kidney Disease Epidemiology Collaboration (CKD‐EPI) formula for the Japanese population.^23^, ^24^ In addition, the eGFR_CRE + CYS_ from the Japanese equation^24^, ^25^ was derived from both serum creatinine and cystatin C values. The other variables measured were systolic blood pressure (SBP), diastolic blood pressure (DBP) and body weight. To determine predicted 24‐hour urinary sodium and potassium at baseline, we used the Tanaka formula:^26^

24‐hour urinary sodium (Na) (mmol/d) = 21.98 × (Naspot [Spot urinary sodium]/Crspot [Spot urinary creatinine] × PrUCr24h [predicted 24‐hour urinary creatinine])^0.392^, urinary potassium (K) (mmol/d) = 7.59 × (Kspot [Spot urinary potassium]/Crspot × PrUCr24h)^0.431^, PrUCr24h = 14.89 × weight (kg) + 16.14 × height (cm) − 2.04 × age (years) – 2244.45. Estimated daily salt intake (DSI) (g/d) = 24‐hour urinary sodium (mmol/d)/17.

To assess the effects of basal DSI on changes in metabolic variables during the use of TOFO, participants were divided into four groups according to quartiles of basal DSI (1st quartile, DSI ˂7.9; 2nd quartile, 7.9 to ˂9.3; 3rd quartile, 9.3 to ˂11.0; 4th quartile, ≥11.0). Adjusted assessments of HbA1c, FPG, sodium, potassium, body weight, SBP, DBP, eGFR_MDRD_, BNP and ACR were analysed using an analysis of covariance (ANCOVA) model, with the baseline values age, sex and eGFR_MDRD_ as covariates to determine changes across quartiles. In the evaluation of adjusted assessments of cystatin C, eGFR_CKD‐EPI_ and **eGFR**_**CRE + CYS**_, changes were analysed using the ANCOVA model, with baseline values, age and sex as covariates. The adjusted assessment of creatinine was also analysed, using the ANCOVA model with baseline values, age and sex as covariates across quartiles of DSI.

Changes in eGFR_MDRD_ were assessed in participants receiving, and in those not receiving, renin‐angiotensin system (RAS) inhibition drugs (ARB and/or ACE inhibitor [ACEI]) as concomitant antihypertensive therapy and in participants with or without hyperfiltration. Participants were divided into two groups according to whether they received RAS inhibition drugs and into another two groups according to basal eGFR_MDRD_ (without hyperfiltration [eGFR_MDRD_ < 120 mL/min/1.73m^2^]; with hyperfiltration [eGFR_MDRD_ = > 120]).

Correlations between change in eGFR_MDRD_ at Weeks 4 and 52 and baseline variables were analysed by Pearson's product‐moment correlation coefficients. Correlations between changes in eGFR_MDRD_ and blood pressure at Weeks 4 and 52 and baseline DSI were investigated in the entire participant population and in those using or not using RAS inhibition drugs as concomitant antihypertensive therapy. Correlations between changes in eGFR_MDRD_ and blood pressure and body weight at Week 4 were also analysed in the entire participant population and in each quartile group according to DSI. Finally, correlations between changes in eGFR_MDRD_ and changes in HbA1c from Week 4 to Week 52 were investigated.

To identify baseline clinical factors that might independently influence changes in eGFR_MDRD_ and creatinine level at weeks 4 and 52, 14 variables at baseline (dosage of TOFO, use of RAS inhibition drugs as concomitant antihypertensive therapy, age, sex, duration of diabetes, HbA1c, DBP, BNP, BMI, uric acid, eGFR_MDRD_, creatinine, DSI and ACR <30 mg/g Cre [vs ACR ≥30 mg/g Cre]) were initially identified based on clinical considerations, although eGFR_MDRD_ and creatinine were adjusted for each other. Moreover, to identify clinical factors that might independently influence changes in eGFR_MDRD_ from Week 4 to Week 52, 12 variables (dosage of TOFO, use of RAS inhibition drugs as concomitant antihypertensive therapy, age, sex, duration of diabetes, DSI, ACR <30 mg/g Cre [vs ACR ≥30 mg/g Cre]**,** and variables at week 4 [HbA1c, DBP, BMI, uric acid**,** and eGFR_MDRD_]) were initially identified based on clinical considerations. Finally, to identify clinical factors that might independently influence eGFR_MDRD_ changes from Week 52 to Week 54, 13 variables (dosage of TOFO, use of RAS inhibition drugs as concomitant antihypertensive therapy, age, sex, duration of diabetes, DSI, ACR <30 mg/g Cre [vs ACR ≥30 mg/g Cre] and variables at Week 52 [HbA1c, DBP, BNP, BMI, uric acid, and eGFR_MDRD_]) were initially identified based on clinical considerations.

For each group, participants' demographics were summarized with appropriate descriptive statistics (means and standard deviation [SD] for continuous variables and counts and percentages for categorical variables). Additionally, differences in between‐group assessments were analysed using ANOVA and chi‐square test. Differences from baseline, from Week 4 to Week 52, and from Week 52 to Week 54 were analysed using the one sample *t* test. In this study, all HbA1c values are presented using the National Glycohemoglobin Standardization Program units. The (two‐sided) significance level for each test was 0.05.

## 3 RESULTS

**Figure 1:**
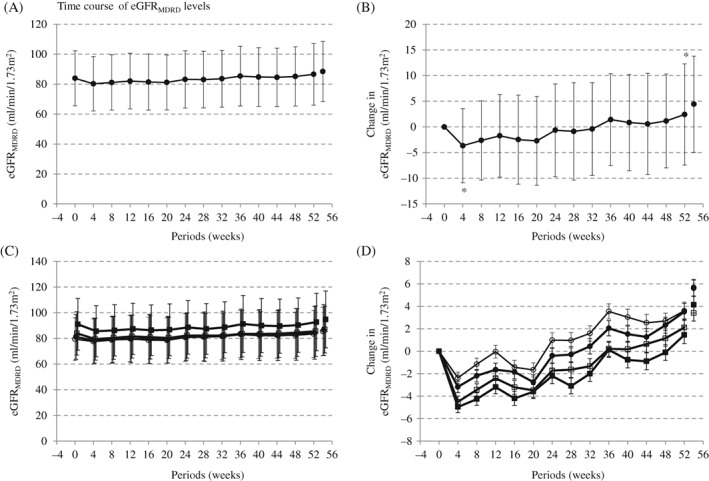
Time course of eGFR_MDRD_ levels. A, Time course of eGFR_MDRD_. B, Changes eGFR_MDRD_. Mean (standard deviation). *P < 0.001 vs baseline, One sample *t* test vs baseline. C, Time course of eGFR_MDRD_ according to quartiles of baseline estimated daily salt intake. (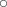, Quartile 1; 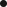, Quartile 2; 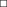, Quartile 3, 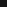, Quartile 4). Mean (standard error). D, Changes in eGFR_MDRD_ according to quartiles of baseline estimated daily salt intake. (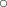, Quartile 1; 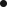, Quartile 2; 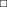, Quartile 3, 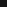, Quartile 4). Least square mean (standard error) adjusted by baseline eGFR_MDRD_ values, age and sex

This pooled analysis included 775 participants (66% men) who were receiving TOFO (Table S1). Mean age, HbA1c, BMI, eGFR_MDRD_ and median estimated DSI were 58.5 years, 8.1%, 25.6 kg/m^2^, 83.9 mL/min/1.73 m^2^, and 9.3 g/d, respectively (Table 1). Minimum and maximum DSI were 3.6 and 19.7 g/d, respectively. During the 52 weeks of treatment with TOFO, the eGFR_MDRD_ of all participants significantly dipped at Week 4 (mean: −3.7 mL/min/1.73 m^2^), gradually increased from Week 4 to Week 52 (+6.1 mL/min/1.73 m^2^) (Figure 1 and Figure S1), and significantly increased from baseline to Week 52 (+2.4 mL/min/1.73 m^2^). Two weeks after termination of TOFO therapy, eGFR_MDRD_ further increased from Week 52. We also observed an inverse time course in serum creatinine levels compared to eGFR_MDRD_ (Figure S2).

**Table 1:** Baseline characteristics according to quartilesof basal estimated daily salt intake

	Basal estimated daily salt intake					
	ALL	Quartile 1	Quartile 2	Quartile 3	Quartile 4	P Across quartiles
N	775	193	194	194	194	
Age (y)	58.5 (10.5)	59.5 (10.5)	59.1 (10.4)	59.2 (9.6)	56.1 (11.2)	0.004
Sex, men / women, n (%)	512 (66.1) / 263 (33.9)	124 (64.2) / 69 (35.8)	135 (69.6) / 59 (30.4)	134 (69.1) / 60 (30.9)	119 (61.3) / 75 (38.7)	0.254
TOFO^a^ 20 mg / 40 mg, n (%)	235 (30.3) / 540 (69.7)	60 (31.1) / 133 (68.9)	58 (29.9) / 136 (70.1)	137 (70.6) / 57 (29.4)	60 (30.9) / 134 (69.1)	0.980
Body weight (kg)	68.4 (14.2)	66.8 (14.4)	66.6 (13.3)	67.8 (13.8)	72.5 (14.6)	<0.001
Body mass index (kg/m2)	25.6 (4.3)	25.3 (4.5)	25.1 (3.7)	25.3 (4.2)	26.7 (4.8)	<0.001
HbA1c (mmol/mol)	64.5 (9.9)	64.5 (10.5)	65.1 (10.5)	63.1 (9.4)	65.3 (9.1)	0.120
HbA1c (%)	8.0 (0.9)	8.0 (1.0)	8.1 (1.0)	7.9 (0.9)	8.1 (0.8)	0.120
Fasting plasma glucose (mmol/L)	8.9 (2.1)	9.1 (2.1)	8.9 (2.1)	8.7 (1.9)	9.0 (2.1)	0.396
Fasting plasma glucose (mg/dL)	160.9 (37.0)	163.3 (37.5)	160.7 (38.3)	157.3 (34.6)	162.3 (37.2)	0.396
Sodium (meq/L)	139.3 (1.9)	139.4 (1.9)	139.2 (1.9)	139.3 (2.1)	139.2 (1.8)	0.627
Potassium (meq/L)	4.2 (0.3)	4.2 (0.3)	4.2 (0.3)	4.1 (0.3)	4.2 (0.3)	0.034
Systolic blood pressure (mm Hg)	130.3 (14.1)	128.7 (14.5)	130.1 (13.7)	130.8 (13.7)	131.8 (14.2)	0.188
Diastolic blood pressure (mm Hg)	77.3 (10.2)	76.8 (10.2)	76.3 (11.0)	77.3 (10.0)	78.9 (9.5)	0.069
Concomitant antihypertensive drugs (%)	366 (47.2)	90 (46.6)	83 (42.8)	96 (49.5)	97 (50.0)	0.465
ARB, n (%)	275 (35.5)	65 (33.7)	64 (33.0)	76 (39.2)	70 (36.1)	0.577
ACEI^b^, n (%)	18 (2.3)	3 (1.6)	6 (3.1)	4 (2.1)	5 (2.6)	0.767
ARB and or ACEI^b^, n (%)	291 (37.6)	67 (34.7)	70 (36.1)	79 (40.7)	75 (38.7)	0.6209
CCB^c^, n (%)	218 (28.1)	51 (26.4)	46 (23.7)	52 (26.8)	69 (35.6)	0.059
Beta‐blockers, n (%)	27 (3.5)	10 (5.2)	7 (3.6)	6 (3.1)	4 (2.1)	0.406
Diuretics, n (%)	64 (8.3)	22 (11.4)	13 (6.7)	13 (6.7)	16 (8.3)	0.309
Duration of diabetes mellitus (y)	7.1 (5.9)	7.1 (6.1)	6.9 (5.9)	7.8 (6.3)	6.8 (5.4)	0.389
Creatinine (mg/dL)	0.71 (0.16)	0.73 (0.15)	0.74 (0.16)	0.71 (0.17)	0.66 (0.17)	<0.001
Cystatin C (mg/L)	0.72 (0.15)	0.74 (0.14)	0.74 (0.16)	0.71 (0.13)	0.70 (0.16)	0.005
eGFRMDRDd (mL/min/1.73 m2)	83.9 (18.4)	79.5 (16.7)	81.2 (17.5)	83.9 (16.9)	91.0 (20.1)	<0.001
eGFRCKD‐EPI^d^ (mL/min/1.73 m2)	84.5 (11.5)	83.1 (10.2)	82.8 (11.7)	83.9 (11.1)	88.3 (12.3)	<0.001
eGFRCRE+CYS^d^ (mL/min/1.73 m2)	97.7 (15.0)	94.9 (14.1)	95.8 (16.1)	98.3 (12.8)	102.0 (15.8)	<0.001
eGFRMDRD^d^≥120 mL/min/1.73 m2, n (%)	30 (3.9)	3 (1.6)	6 (3.1)	7 (3.6)	14 (7.2)	0.0365
Uric acid (mg/dL)	5.1 (1.3)	5.3 (1.3)	5.1 (1.4)	5.1 (1.2)	4.8 (1.3)	0.012
BNP^e^ (pg/mL)	15.6 (17.4)	13.2 (12.2)	14.4 (16.7)	15.3 (13.8)	19.6 (23.8)	0.002
ACRf (mg/g Cre)	16.4 (9.0–46.0)	14.2 (8.5–37.1)	16.2 (8.8–47.1)	15.5 (9.3–43.2)	21.5 (9.5–63.5)	0.028
ACR^f^ = >30 mg/g Cre, n (%)	255 (32.9)	52 (26.9)	63 (32.5)	60 (30.9)	80 (41.2)	0.023
Urinary Na excretion (mmol/d)	157.5 (134.3–186.8)	118.0 (104.6–126.6)	147.1 (140.1–152.8)	170.8 (163.8–177.7)	205.8 (194.2–227.6)	<0.001
Urinary K excretion (mmol/d)	61.1 (52.7–68.6)	51.8 (46.3–59.2)	59.1 (52.0–65.8)	62.6 (55.4–68.8)	69.5 (63.5–75.7)	<0.001
Estimated daily salt intake (g/d)	9.3 (7.9–11.0)	6.9 (6.2–7.5)	8.7 (8.2–9.0)	10.1 (9.6–10.5)	12.1 (11.4–13.4)	<0.001

*Note*: Data are expressed as mean (SD). Urinary indices are expressed as median (interquartile range). Estimated daily salt intake is expressed as median (interquartile range). Analyses were performed by ANOVA (continuous variables) and chi‐squared test (categorical variables). Urinary indices were analyzed using Kruskal‐Wallis.

a Tofogliflozin.

b ACE inhibitor.

c Calcium channel blocker.

d Estimated glomerular filtration rate.

e Brain natriuretic peptide.

f Urine albumin‐to‐creatinine ratio.

Participants were divided into four groups according to quartiles of estimated basal DSI (Table 1). Age was greater, and BMI, eGFRs (eGFR_MDRD_, eGFR_CKD‐EPI_, eGFR_CRE + CYS_) and the proportion of participants with albuminuria (ACR = >30 mg/g Cre) were higher, based on increased basal DSI, while glycemic status and blood pressure were consistent among all quartiles. Changes in variables, glycaemic status, body weight and blood pressure, at both Weeks 4 and 52 were consistent across quartiles (Table S2). Moreover, differences in BNP level and urine ACR were insignificant across quartiles at Week 52. On the contrary, at Week 4, the reductions in eGFR_MDRD_ and eGFR_CKD‐EPI_ were higher, based on increased basal DSI. At Week 52, the increase in eGFR_MDRD_ and eGFR_CKD‐EPI_ tended to be smaller, according to the increased basal DSI, while the reduction in eGFR_CRE + CYS_ and increase in cystatin C were greater, based on increased basal DSI.

At Week 4, baseline HbA1c, fasting plasma glucose, SBP, BNP, urinary sodium and potassium excretion, and DSI were significantly correlated with the change in eGFR_MDRD_ (Table S3). There were significant differences in the change in eGFR_MDRD_ at Week 4 according to dosage of TOFO, use of concomitant antihypertensive drugs including ARB and RAS inhibition drugs, and ACR category (<30 mg/g Cre or ≥ 30) (Table S4). At Week 52, age, baseline HbA1c, fasting plasma glucose, cystatin C and urinary sodium excretion, and DSI were correlated with change in eGFR_MDRD_ (Table S3). At Week 52 there were significant differences in the change in eGFR_MDRD,_ according to the use of concomitant antihypertensive drugs, including the use of ARB (Table S4).

Change in eGFR_MDRD_ at Week 4 was not correlated with change in DBP (r = 0.01; *P* = 0.780) in the entire participant group, while change in eGFR_MDRD_ at Week 4 was significantly correlated with change in SBP (r = 0.10; *P* = 0.004) and with change in body weight (r = 0.20; *P* < 0.001). There was not a significant correlation between change in eGFR_MDRD_ from Week 4 to Week 52 and change in HbA1c (r = 0.05; *P* = 0.163).

**Figure 2:**
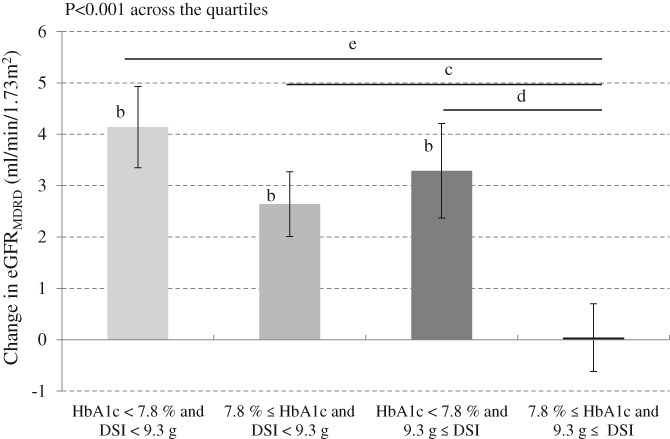
Changes in eGFR_MDRD_ at Week 52 according to median values of baseline HbA1c and estimated daily salt intake. ^a^ *P* < 0.01, ^b^ *P* < 0.001 vs baseline, ^c^ *P* < 0.05, ^d^ *P* < 0.01, ^e^ *P* < 0.001 vs HbA1c ≥7.8% and DSI ≥9.3 g. One sample *t* test

Multivariable analysis demonstrated that higher basal DSI and HbA1c levels, and use of RAS inhibition drugs, were negatively correlated with changes in eGFR_MDRD_ at Week 4 (Table 2), but were positively correlated with changes in creatinine levels (Table S5). At Week 52, higher basal DSI and HbA1c levels, and use of RAS inhibition, drugs were negatively correlated with changes in eGFR_MDRD_, but were positively correlated with changes in creatinine levels. Both lower glycaemic status and DSI at baseline were correlated with the greater increase in eGFR_MDRD_ at Week 52 (Figure 2).

**Table 2:** Baseline predictors for influencing the change in eGFRmdrdat week 4 and week 52

Change in eGFRmdrdat week 4		
Factors	Regression coefficient	*P*
BNP (higher 1 pg/mL)	−0.04	0.0099
eGFR (higher 1 mL/min/1.73 m^2^)	−0.08	<0.001
Estimated daily salt intake (higher 1 g/d)	−0.47	<0.001
HbA1c (higher 1%)	−0.59	0.0320
Tofogliflozin 40 mg (vs. 20 mg)	−1.07	0.0464
Use of RAS inhibition drugs (yes)	−1.22	0.0176

Change in eGFRmdrdat week 52		
Factors	Regression coefficient	*P*
Age (higher 1 year)	−0.07	0.0373
Estimated daily salt intake (higher 1 g/d)	−0.40	0.0104
HbA1c (higher 1%)	−1.69	<0.001
Use of RAS inhibition drugs (yes)	−2.25	0.0034

*Note*: Factors remained through stepwise variable selection with *P* < 0.05. Potential baseline predictors were dosage of tofogliflozin, use of RAS inhibition drugs (ARB and/or ACEI),age, sex, duration of diabetes, HbA1c, DBP, BNP, BMI, uric acid, eGFRmdrd, DSI levels, and ACR <30 mg/g Cre (vs. ACR = >30 mg/g Cre).

## 4 DISCUSSION

The present study is the first to show an independent correlation between basal DSI and changes in eGFR_MDRD_ at both Weeks 4 and 52 during treatment with the SGLT2i, TOFO, in patients with T2D. We also found that basal DSI did not correlate with improved hyperglycaemia, decreased body weight and blood pressure, and changes in ACR. Moreover, participants with both lower basal HbA1c and DSI experienced greater increases in eGFR_MDRD_ from baseline to Week 52. Based on these findings, we concluded that changes in eGFR_MDRD_ as the result of TOFO indicated the attenuated glomerular relative‐hyperfiltration and subsequent improved renal function, which might be attributed to the handling of reabsorption of, not only urinary glucose, but also sodium, through SGLT2 in the proximal tubule.

eGFR_MDRD_ levels initially dipped at Week 4, which is consistent with results of previous studies.^18^, ^27^ However, the clinical factors that influenced the initial reduction in eGFR_MDRD_ remain to be elucidated. In our participants, the basal eGFR_MDRD_ increased with higher levels of basal DSI, suggesting that the proportion of participants with glomerular relative‐hyperfiltration might be higher following increases in DSI across quartiles. Multivariable analyses also indicated that basal DSI was correlated with eGFR_MDRD_ and changes in creatinine levels, independent of basal eGFR_MDRD_, creatinine level, HbA1c level and use of RAS inhibition drugs. Therefore, these differences in the degree of initial dip in eGFR_MDRD_ as the result of TOFO was attributed to the correction of TGF action via SGLT2i. These findings suggested that salt intake‐induced glomerular relative‐hyperfiltration might be caused by SGLT2 in the proximal tubule and that the inhibition of SGLT2 might attenuate it in patients with T2D.

Our results also indicated that the change in eGFR_MDRD_ at Week 4 was not correlated with changes in DBP, but was significantly correlated with changes in SBP in the entire participant population. However, those correlations were weak. Further, the change in eGFR_MDRD_ was not correlated with changes in blood pressure, according to results in each of the DSI quartile groups. Therefore, the contribution of changes in blood pressure to the change in eGFR_MDRD_ at Week 4 might be small. We further indicated that baseline BNP might be a predictor of the change in eGFR_MDRD_ at Week 4, according to results of multivariable analysis, although this observation might not apply to the change in eGFR_MDRD_ at Week 52. Higher BNP levels, suggesting fluid retention, might cause the greater reduction in eGFR_MDRD_ as the result of TOFO at Week 4. Thus, fluid loss soon after TOFO treatment might contribute, in part, to the reduction in eGFR_MDRD_ at Week 4. That change in eGFR_MDRD_ at Week 4 was significantly correlated with change in body weight provides support that fluid loss might be associated, in part, with the reduction in eGFR_MDRD_ at Week 4.

We also observed a gradual increase in eGFR_MDRD_ from Week 4 to Week 52, irrespective of basal DSI. This recovery of eGFR_MDRD_ from the initial decrease was consistent with that shown in previous studies.^18^, ^27^ However, in this study, a significant increase in eGFR_MDRD_ from baseline to Week 52 was observed. Results of multivariable analysis also indicated that basal DSI was not correlated, but HbA1c levels at Week 4 were negatively correlated with recovery of eGFR_MDRD_ (Table S6), suggesting that the lower levels of HbA1c soon after TOFO treatment might have contributed to that recovery. Further, change in eGFR_MDRD_ from Week 4 to Week 52 was not correlated with change in HbA1c. These results suggested that, not only the correction of salt intake‐induced glomerular relative‐hyperfiltration, but also the well‐controlled hyperglycaemia before and soon after TOFO administration, might contribute to the recovery of and increase in eGFR_MDRD_ after the initial dip. Prolonged hyperglycaemia directly induces mesangial expansion and injury,^28^ and also causes cellualar dysfunction in the proximal tubular cells as the result of hyperglycemia‐induced excessive glucose reabsorption.^29^ Thus, the recovery of eGFR_MDRD_ from the initial decrease might be attributed to, not only attenuation of glomerular relative‐hyperfiltration, but also the baseline renal status induced by a sustained well‐controlled glycaemic status.

We observed changes in eGFR_MDRD_ during TOFO treatment, according to whether participants used RAS inhibition drugs (Figure S3). There were significant differences in the change in eGFR_MDRD_ at both Week 4 and Week 52, according to use of RAS inhibition drugs. Results of multivariable ananysis indicated that the use of RAS inhibition drugs as concomitant antihypertensive medication might be negatively correlated with change in eGFR_MDRD_ at Weeks 4 and 52. Therefore, the use of RAS inhibition drugs as concomitant antihypertensive medication might contribute to the effects of TOFO treatment on eGFR_MDRD_ levels, independent of basal DSI. The preferential effect of SGLT2 inhibition on afferent renal arteriolar resistance was reported to be important in reducing intraglomerular pressure,^30^ while the effects of RAS inhibition on efferent renal arteriolar resistance might be thought to be important also in reducing intraglomerular pressure. Therefore, the combination of SGLT2 inhibition drugs and RAS inhibition drugs might contribute to further correction of increased intraglomerular pressure, leading to a delay in the development and progression of diabetic kidney disease.^30^, ^31^ Our results suggested that the use of RAS inhibition drugs might independently contribute to a further reduction in eGFR_MDRD_, followed by decreased intraglomerular pressure. On the contrary, our findings also supported the association of basal DSI with change in eGFR_MDRD_ at Weeks 4 and 52, independent of the use of RAS inhibition drugs. However, the monitored period of TOFO treatment was limited and histological confirmation was not performed to evaluate structural changes in the kidney. Therefore, further investigation will be needed to evaluate the effects of RAS inhibition drugs on changes in eGFR as the result of long‐term SGLT2i treatment.

From our results, we could not explain the association of the Na+/H + ‐exchanger 3(NHE3), which contributed to reabsorption of urinary sodium, with the effects of long‐term TOFO treatment on eGFR_MDRD_. SGLT2 is co‐expressed with NHE3, which reabsorbs approximately 30% of filtered sodium. Recent studies provide evidence that SGLT2 may be functionally linked to NHE3, such that SGLT2 inhibition may also inhibit NHE3 in the proximal tubule.^4^, ^32^, ^33^ Our study indicated that baseline DSI, which was estimated from urinary sodium excretion, might independently influence changes in eGFR_MDRD_ during long‐term TOFO treatment. The interaction between SGLT2 and NHE3, and change in the reabsorption of sodium leading to increased delivery of sodium to the macula densa after SGLT2 inhibition, could be relevant in explaining the effect of SGLT2i on eGFR. Further investigations are required to clarify those interactions and their effect on eGFR as the result of SGLT2i treatment.

Two weeks after termination of treatment with TOFO, eGFR_MDRD_ levels had further increased from Week 52, which was consistent with previous reports.^18^ These results supported the opinion that, with long‐term administration of SGLT2i, the attenuation of glomerular relative‐hyperfiltration might be maintained. Recently, Cherney et al. reported that, 30 days after termination of treatment with empagliflozin, a greater reduction in urine ACR was maintained with emphagliflozin than with a placebo.^34^ In our study, the positive correlation between change in eGFR_MDRD_ and urine ACR from Week 52 to Week 54 was observed in participants with albuminuria (Figure S4). This result suggested that attenuation of glomerular relative‐hyperfiltration might contribute, in part, to the reduction in urine ACR. The report by Cherney et al. also suggested that long‐term administration of empagliflozin might contribute to functional improvement of the kidney.^34^ Results of our correlation analysis indicated that increased eGFR_MDRD_ during the termination period was not significantly correlated with the initial decrease, which might be caused by the attenuation of glomerular relative‐hyperfiltration (Figure S5). Moreover, DSI was not significantly associated with increased eGFR_MDRD_ during the termination period, based on results of multivariable analysis (Table S7). These findings suggested that increased eGFR_MDRD_ after the termination of TOFO treatment might be caused, not only by the released attenuation of glomerular relative‐hyperfiltration, but possibly by other factors as the result of long‐term use of the medication, such as functional changes in the kidney.

Although study participants had a mean eGFR_MDRD_ value at baseline of 83.9 mL/min/1.73m^2^, 30 of these participants had eGFR_MDRD_ levels ≥120 mL/min/1.73m^2^ (hyperfiltration). The proportion of participants with hyperfiltration tended to increase in association with increased basal DSI. We further investigated the effects on eGFR_MDRD_, according to whether participants had hyperfiltration during the study period (Figure S6). Reduction in eGFR_MDRD_ at Week 4 was greater in participants with hyperfiltration than in those without hyperfiltration, while changes in eGFR_MDRD_ from Week 4 to Week 52, from baseline to Week 52, and from Week 52 to Week 54 were identical between participants with and without hyperfiltration. Changes in eGFR_MDRD_ during TOFO treatment were similar between participants with and without hyperfiltration, with the exception of the initial dip in eGFR_MDRD._ However, further study involving a large number of participants with hyperfiltration is needed to clarify the effects of basal DSI on eGFR as the result of SGLT2i treatment.

Current approaches to preventing kidney complications in patients with diabetes have focused on lowering blood pressure, HbA1c, body weight, albuminuria and cholesterol, which have been shown to reduce the risk of cardiovascular disease and deterioration of kidney function.^35^, ^36^ Our analysis also revealed decreased blood pressure, HbA1c and body weight at Weeks 4 and 52. The reductions in BP according to basal DSI quartiles were similar, regardless of the use of RAS inhibition drugs as concomitant antihypertensive medication. Therefore, these results might not indicate a clear association between baseline DSI and reductions in blood pressure after long‐term TOFO treatment. TOFO was previously reported to reduce urine ACR and the N‐acetyl‐beta‐D‐glucosaminidase/creatinine ratio in patients with T2D with micro‐ and macroalbuminuria.^37^ In this study, less‐controlled glycaemic status at baseline was associated with subsequent increase in eGFR_MDRD_ as the result of TOFO treatment. Interestingly, independent of glycaemic status, lower salt intake at baseline was also associated with subsequent increase in eGFR_MDRD._. Our findings suggested that basal DSI, in addition to correlation with glycaemic control, correlates with eGFR_MDRD_ as the result of long‐term TOFO treatment in participants with T2D, independent of the use of RAS inhibition drugs as concomitant antihypertensive treatment. Thus, an appropriate dietary approach to therapy should be considered before initiation of treatment with SGLT2is. On the contrary, glomerular relative‐hyperfiltration induced by higher DSI might be attenuated by TOFO treatment, leading to a further reduction in eGFR_MDRD_ levels. More studies are required to clarify whether these differences in results regarding the changes in eGFR_MDRD_ that are influenced by basal DSI may contribute to clinically meaningful reductions in the development and progression of diabetic kidney diseases in patients with T2D. Unfortunately, we could not monitor changes in urinary sodium excretion in order to estimate changes in salt intake during TOFO treatment. Sustained lower‐controlled salt intake may contribute to the further increase in eGFR during SGLT2i treatment, but additional investigations will be needed. SGLT2i inhibits the reabsorption of, not only urine glucose, but also sodium, which may have multifactorial effects on the risk factors for cardiovascular disease and deterioration of kidney function. However, dietary approaches may be needed, particularly in monitoring salt intake, to maximize the renal effects of SGLT2i.

We recognize that the estimation of DSI in our study is a serious limitation. Although we performed the analyses according to quartiles of basal DSI, SBP and DBP levels at baseline did not differ among the quartiles of DSI, nor did the proportion of concomitant antihypertensive drugs at baseline. Of note, the use of diuretics coincided with increases in basal DSI, although the use of diuretics might be clinically effective in controlling blood pressure in salt‐sensitive hypertensive participants. Our study was based on pooled analyses from two TOFO studies that had been designed to evaluate the safety and efficacy of TOFO as monotherapy or as an add‐on to other oral antidiabetic agents in T2D patients. Thus, the prescribed antihypertensive drugs might not have been selected according to basal DSI. Among study participants, 47.2% received antihypertensive drugs and 8.3% were received diuretics. We found that the proportion of participants with ACR levels ≥30 mg/g Cre was significantly increased, based on increased basal DSI. In Japanese T2D patients with microalbuminuria, the prevalence of hypertension that was salt sensitive was greater than that in patients without microalbuminuria,^38^ which provides support that our basal DSI levels were correctly estimated. Also, the formula for estimating DSI levels was calculated according to participant age, body weight, and urinary sodium secretion. Higher basal DSI values were more prevalent in younger participants and in those with higher body weight. It can be considered that the formula for estimating DSI in our study is applicable to clinical practice for patients receiving concomitant diuretics.^39^ Of course, further investigation of the effects of DSI on eGFR as the result of SGLT2i treatment, according to the use of concomitant hypertensive drugs, especially diuretics, will be needed. Importantly, measuring and monitoring actual daily urinary sodium excretion and investigating its association with the effect of long‐term SGLT2i treatment would be desirable.

This study has several other limitations. We could not perform a comparison between a placebo and TOFO, and the baseline eGFR_MDRD_ level in almost all participants was less than 120 mL/min/1.73m^2^. We could not measure the actual GFR and variables related to intrarenal haemodynamic function. Histological confirmation was not performed to evaluate structural changes in the kidney. This study was the first to elucidate baseline predictors of changes in eGFR_MDRD_ at Weeks 4 and 52 from the results of multivariable analyses. However, the contribution of these predictors to change in eGFR_MDRD_ according to the multivariable analyses might be small; more studies to determine greater contributions to eGFR as the result of SGLT2i treatment are required. Also, our results were obtained in Japanese participants with T2D, whose DSI might be higher than that in other populations.^40^ Thus, investigation of the impact of salt intake on the kidney as the result of SGLT2i treatment in areas other than those addressed here is needed. Finally, prospective long‐term placebo‐controlled randomized studies with larger cohorts, *in vitro* molecular actions and pharmacological factors are required to confirm conclusions based on the results of this study.

In conclusion, basal DSI was independently correlated with changes in eGFR_MDRD_ after long‐term treatment with an SGLT2i, TOFO, in patients with T2D. Moreover, both low basal HbA1c and DSI levels were associated with a greater increase in eGFR_MDRD_ at Week 52. The effects of the SGLT2i, TOFO, on eGFR might be associated with the handling of, not only glucose, but also sodium, through SGLT2 in the proximal tubule.

## CONFLICT OF INTEREST

K. K. has been an advisor to and has received honoraria for lectures from Astelas, Novo Nordisk Pharma, Sanwa Kagaku Kenkyusho, Takeda, Taisho Pharmaceutical, MSD, Kowa, Kissei, Sumitomo Dainippon Pharma, Novartis, Mitsubishi Tanabe Pharma, Nippon Boehringer Ingelheim, Daiichi Sankyo, and Sanofi. A. Y. is an employee of Kowa Pharmaceutical. H. S. is an employee of Kowa. Y. S. had no conflicts of interest.

## AUTHOR CONTRIBUTIONS

All authors participated in writing the manuscript. K. N. and Y. S. wrote the draft of the manuscript and contributed to the discussion. K. K. contributed to the discussion and reviewed/edited the manuscript. A. Y. researched data and reviewed the manuscript. H. S. had full access to all data in the study and takes responsibility for the integrity of the data and the accuracy of the data analyses. K. K. is a director of data collection. H. S. and K. K. are the guarantors of this work.

## Supporting information


**Figure S1**. eGFR_MDRD_ changes from week 4 to week 52 and from week 52 to week 54.
**Figure S2**. Time course of creatinine levels.
**Figure S3**. Time course of eGFR_MDRD_ levels according to the participants with or without RAS inhibition drugs (ARB and or ACEI) as concomitant antihypertensive drugs.
**Figure S4**. Correlation between eGFR_MDRD_ and urine ACR changes from week 52 to week 54.
**Figure S5**. Correlation between eGFR_MDRD_ changes from baseline to week 4 and from week 52 to week 54.
**Figure S6**. Time course of eGFR_MDRD_ levels according to participants whose basal eGFR_MDRD_ levels were < 120 mL/min/1.73m^2^ and = > 120.

**Table S1**. Integrated analysis of two clinical studies.
**Table S2**. Change in variablesat week 4 and week 52 according to quartiles of daily salt intake.
**Table S3**. Baseline predictors for the change in serum creatinine levels at week 4 and week 52.
**Table S4**. Predictors for change in eGFRMDRDfrom week 4 to week 52.
**Table S5**. Predictors ofchange in eGFRMDRDfrom week 52 to week54.
**Table S6**. Correlations between the change in eGFRMDRDat week 4 and week 52 and baseline continuous variables.
**Table S7**. Change in eGFRMDRDat week 4 and week 52 according to baseline categorical variables.
