# Supplementary material for: Renal effects of a sodium‐glucose cotransporter 2 inhibitor, tofogliflozin, in relation to sodium intake and glycaemic status
Source: Diabetes Obes Metab. 2019 May 6;21(7):1715–24. doi: 10.1111/dom.13731 (PMC6619387; doi:10.1111/dom.13731)
Supplement: Supplementary file 1 — Figure S1. eGFRMDRD changes from week 4 to week 52 and from week 52 to week 54. Figure S2. Time course of creatinine levels. Figure S3. Time course of eGFRMDRD levels according to the participants with or without RAS inhibition drugs (ARB and or ACEI) as concomitant antihypertensive drugs. Figure S4. Correlation between eGFRMDRD and urine ACR changes from week 52 to week 54. Figure S5. Correlation between eGFRMDRD changes from baseline to week 4 and from week 52 to week 54. Figure S6. Time course of eGFRMDRD levels according to participants whose basal eGFRMDRD levels were < 120 mL/min/1.73m2 and = > 120. [file DOM-21-1715-s001.pdf]

Supplemental Fig 1. $eGFR_{MDRD}$ changes from week 4 to week 52 and from week 52 to week 54

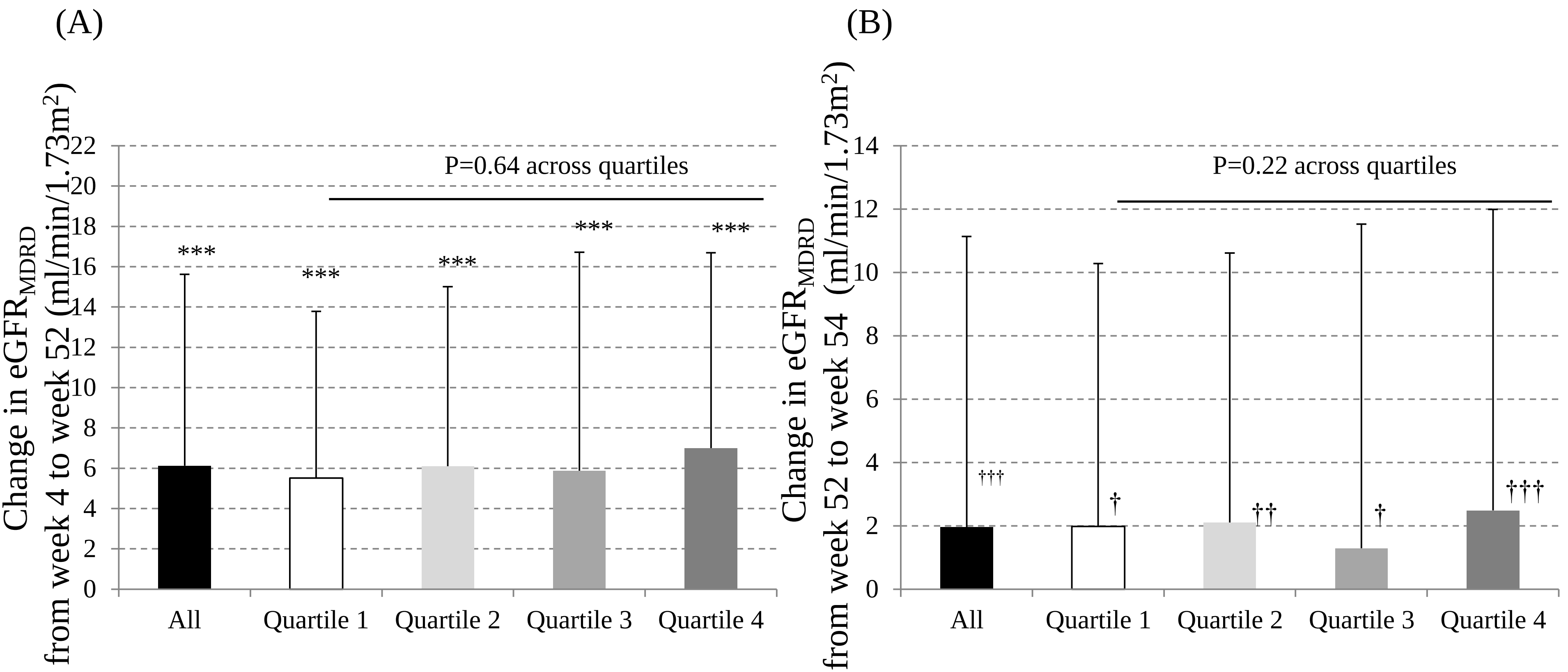

(A) $eGFR_{MDRD}$ changes from week 4 to week 52
Mean (standard deviation)
ANCOVA adjusted by age, sex and $eGFR_{MDRD}$ values at week 4
One sample t-test *** p<0.001 vs. week 4
(B) $eGFR_{MDRD}$ changes from week 52 to week 54
Mean (standard deviation)
ANCOVA adjusted by age, sex and $eGFR_{MDRD}$ values at week 52
One sample t-test † p<0.05, †† p<0.01, ††† p<0.001 vs. week 52

Supplemental Fig 2. Time course of creatinine levels

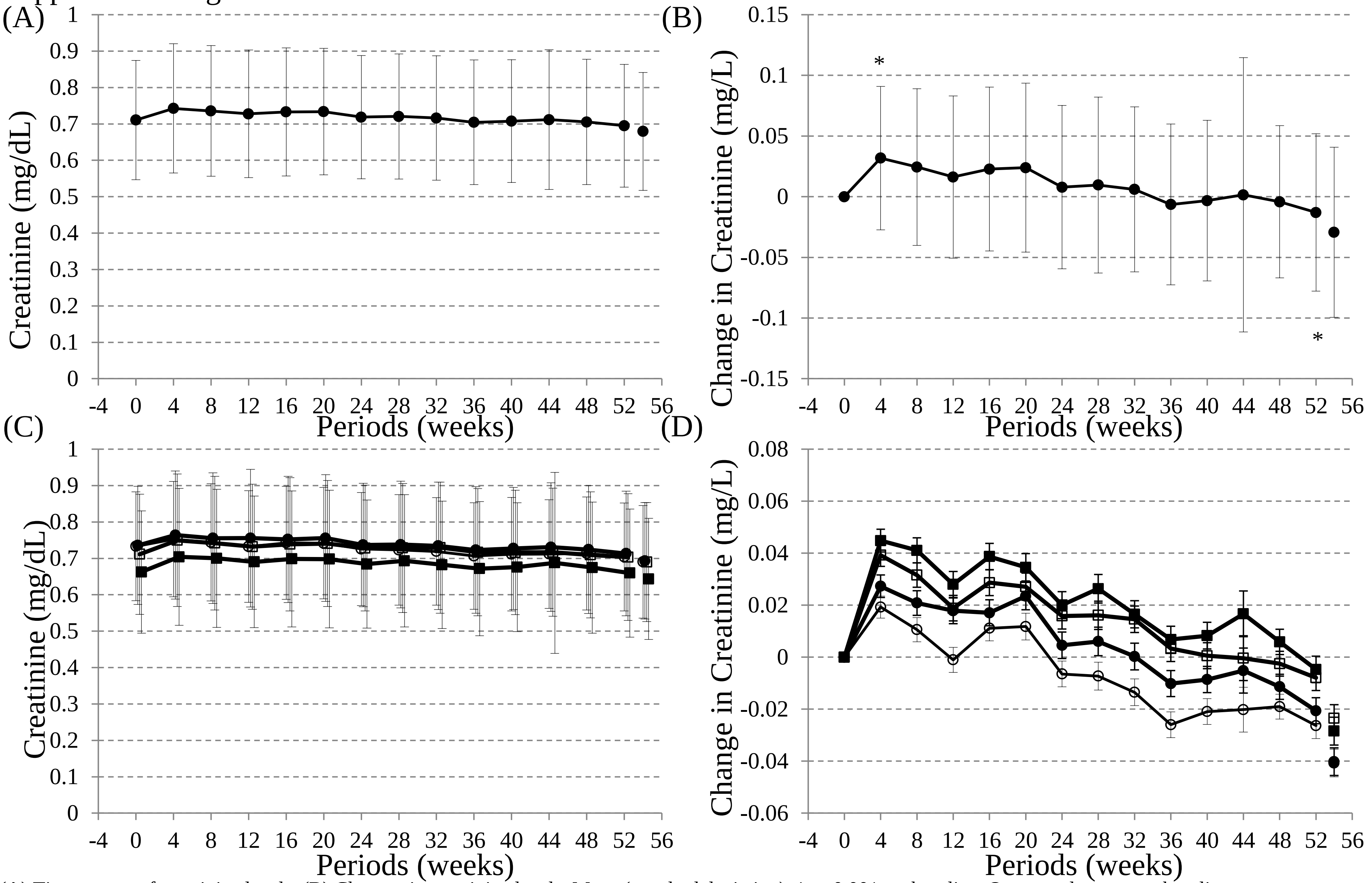

(A) Time course of creatinine levels, (B) Changes in creatinine levels, Mean (standard deviation), * p<0.001 vs. baseline, One sample t-test vs. baseline

(C) Time course of creatinine levels according to quartiles of baseline estimated daily salt intake, (○, Quartile 1; ●, Quartile 2; □, Quartile 3, ■, Quartile 4), Mean (standard error)

(D) Changes in creatinine levels according to quartiles of baseline estimated daily salt intake, (○, Quartile 1; ●, Quartile 2; □, Quartile 3, ■, Quartile 4)
Least square mean (standard error) adjusted by age, sex and baseline creatinine values

Supplemental Fig 3. Correlation between $eGFR_{MDRD}$ and urine ACR changes from week 52 to week 54

(A)

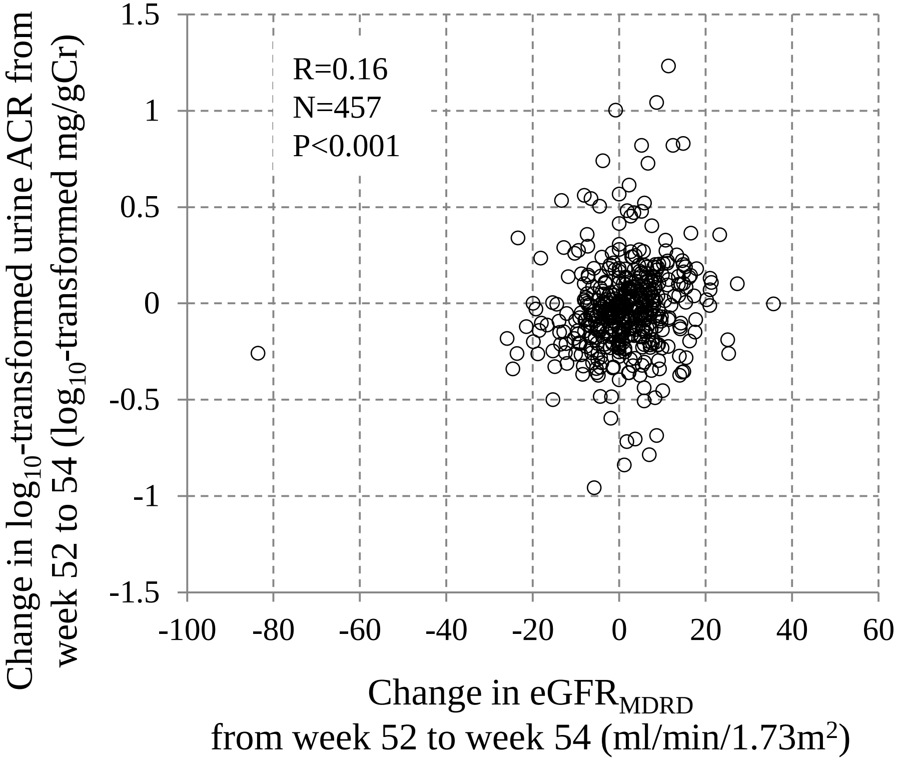

(B)

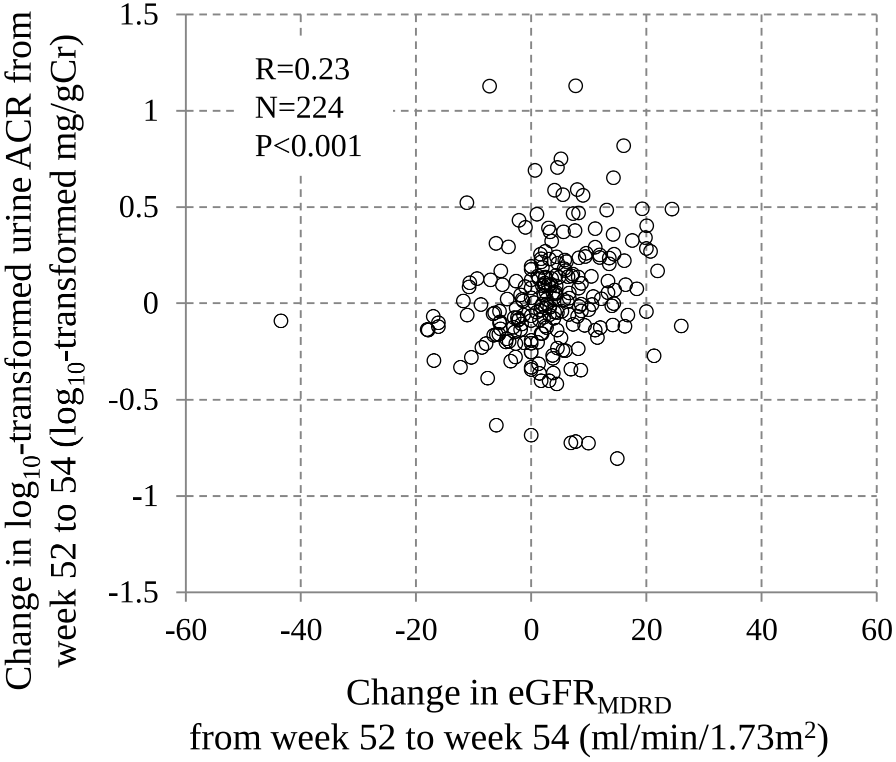

(A) Correlation between $eGFR_{MDRD}$ and urine ACR changes from week 52 to week 54 in participants without albuminuria (ACR <30 mg/g Cre)
(B) Correlation between $eGFR_{MDRD}$ and urine ACR changes from week 52 to week 54 in participants with albuminuria (ACR = >30 mg/g Cre)
Pearson's coefficient

Supplemental Fig 4. Correlation between $eGFR_{MDRD}$ changes from baseline to week 4 and from week 52 to week 54

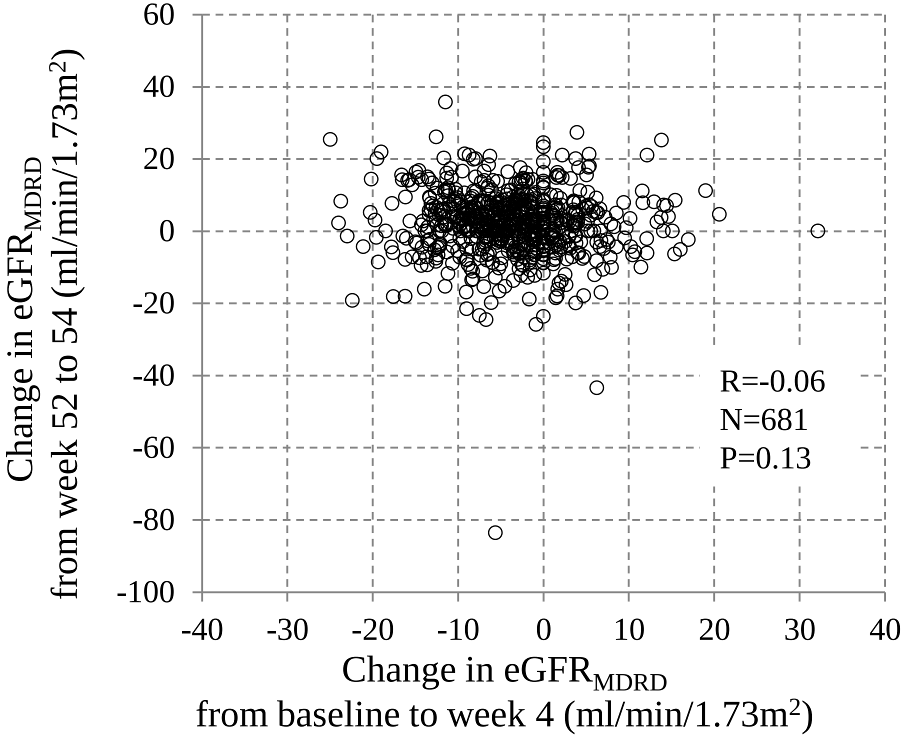

Pearson’s coefficient

Supplemental Fig 5. Time course of $eGFR_{MDRD}$ levels according to participants whose basal $eGFR_{MDRD}$ levels were < 120 ml/min/1.73m^2 and = > 120

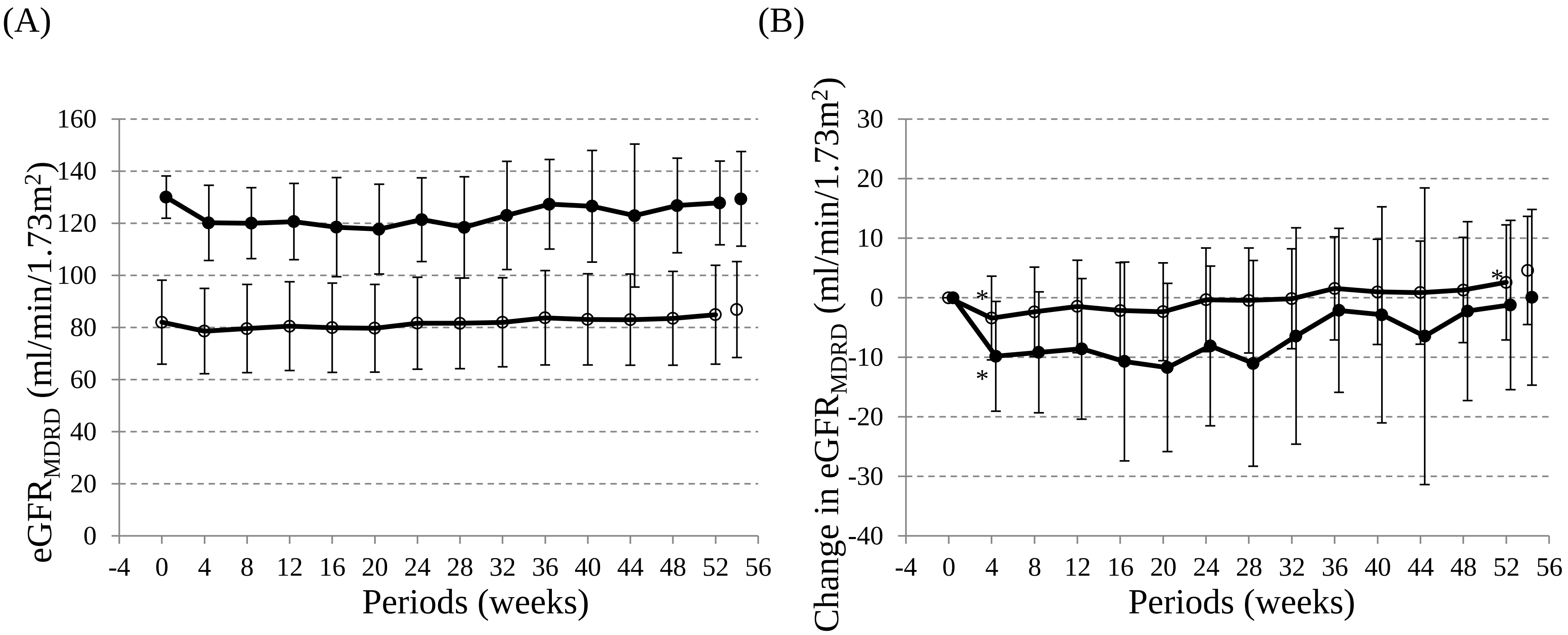

(A) Time course of $eGFR_{MDRD}$ (○, basal $eGFR_{MDRD}$ < 120 ml/min/1.73m^2; ●, eGFR = > 120)
(B) $eGFR_{MDRD}$ changes (○, basal $eGFR_{MDRD}$ < 120 ml/min/1.73m^2; ●, eGFR = > 120)
Mean (standard deviation), * p<0.001 vs. baseline, One sample t-test vs. baseline

Supplemental Fig 6. Time course of $eGFR_{MDRD}$ levels according to participants with or without RAS inhibition drugs (ARB and or ACEI) as concomitant antihypertensive drugs

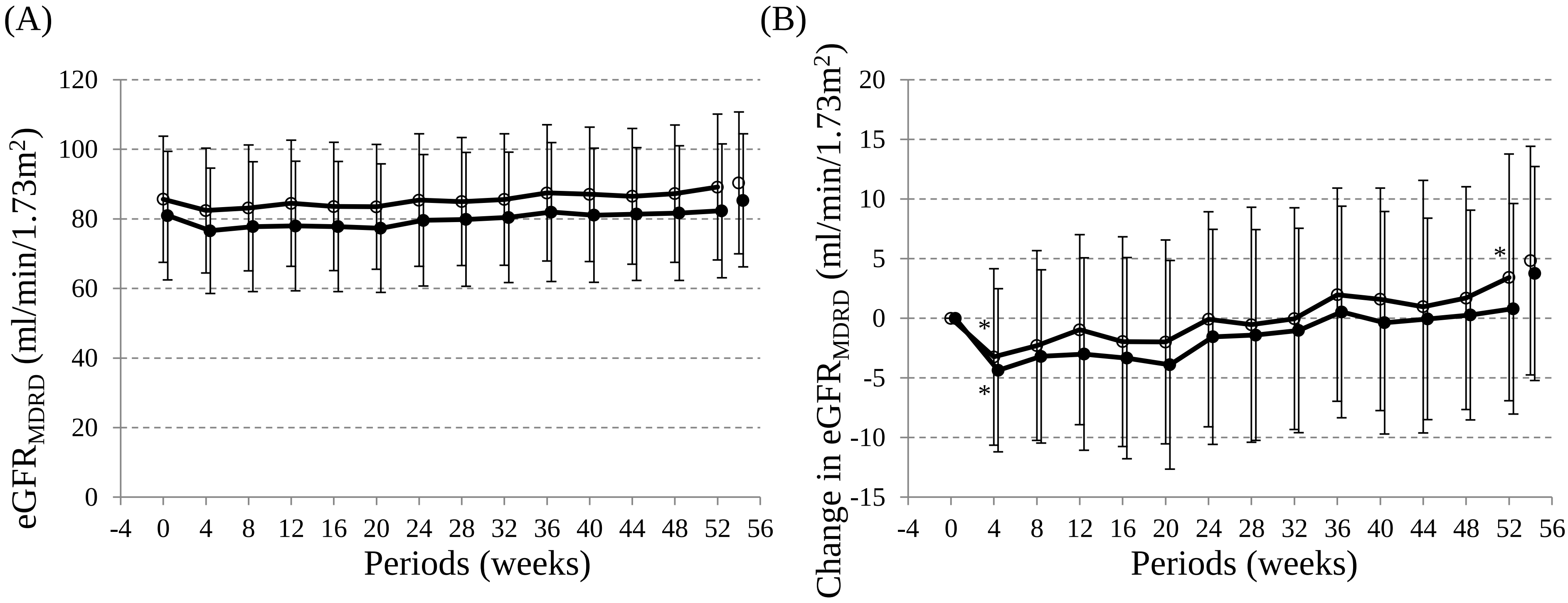

(A) Time course of $eGFR_{MDRD}$ (○, with RAS inhibition drugs; ●, without RAS inhibition drugs)
(B) $eGFR_{MDRD}$ changes(○, with RAS inhibition drugs; ●, without RAS inhibition drugs)
Mean (standard deviation), * $p<0.001$ vs. baseline, One sample t-test vs. baseline
