# Supplementary material for: Renal effects of a sodium‐glucose cotransporter 2 inhibitor, tofogliflozin, in relation to sodium intake and glycaemic status
Source: Diabetes Obes Metab. 2019 May 6;21(7):1715–24. doi: 10.1111/dom.13731 (PMC6619387; doi:10.1111/dom.13731)
Supplement: Supplementary file 2 — Table S1. Integrated analysis of two clinical studies. Table S2. Change in variablesat week 4 and week 52 according to quartiles of daily salt intake. Table S3. Baseline predictors for the change in serum creatinine levels at week 4 and week 52. Table S4. Predictors for change in eGFRMDRDfrom week 4 to week 52. Table S5. Predictors ofchange in eGFRMDRDfrom week 52 to week54. Table S6. Correlations between the change in eGFRMDRDat week 4 and week 52 and baseline continuous variables. Table S7. Change in eGFRMDRDat week 4 and week 52 according to baseline categorical variables. [file DOM-21-1715-s002.pdf]

Supplemental Table 1. Integrated analysis of two clinical studies

Study	Design	Treatment	Dosage (n)	Periods (weeks)	Baseline						
					Sex (Male/Female)	Age (years)	BMI (kg/m^2)	HbA1c (mmol/mol)	HbA1c (%)	$eGFR_{MDRD}$ ($ml/min/1.73m^2$)	Estimated daily salt intake (g/day)
004JP[1]	Open-label, randomized controlled study	Monotherapy	20 mg (63)<br>40 mg (127)	52	126 / 64	58.1<br>(10.8)	25.6<br>(4.5)	62.1<br>(9.9)	7.8<br>(0.9)	83.0<br>(18.2)	9.2<br>(2.4)
005JP[1]	Open-label, randomized controlled study	Add-on therapy (One drug)	20 mg (172)<br>40 mg (413)	52	386 / 199	58.6<br>(10.4)	25.6<br>(4.3)	65.3<br>(9.8)	8.1<br>(0.9)	84.2<br>(18.4)	9.6<br>(2.4)
Total	Two prospective studies	Monotherapy or Add-on Therapy	20 mg (235)<br>40 mg (540)	52	512 / 263	58.5<br>(10.5)	25.6<br>(4.3)	64.5<br>(9.9)	8.0<br>(0.9)	83.9<br>(18.4)	9.5<br>(2.4)

Data are expressed as mean (standard deviation). Abbreviations: HbA1c, glycated hemoglobin; eGFR, estimated glomerular filtration rate.

1 Tanizawa Y et al. Expert Opin Pharmacother. 2014 Apr;15(6):749-66.

Supplemental Table 2. Change in variables at week 4 and week 52 according to quartiles of daily salt intake

	Estimated daily salt intake				
At week 4	Quartile 1	Quartile 2	Quartile 3	Quartile 4	p
N	193	194	194	194	
HbA1c (mmol/mol)	-4.09 (0.27)**	-3.72 (0.27)**	-3.80 (0.27)**	-4.28 (0.27)**	0.41
HbA1c (%)	-0.37 (0.02)**	-0.34 (0.03)**	-0.35 (0.02)**	-0.39 (0.02)**	0.41
Fasting plasma glucose (mmol/L)	-1.59 (0.07)**	-1.45 (0.07)**	-1.35 (0.07)**	-1.52 (0.07)**	0.06
Fasting plasma glucose (mg/dL)	-28.6 (1.2)**	-26.1 (1.2)**	-24.3 (1.2)**	-27.3 (1.2)**	0.06
Sodium (mEq/L)	0.42 (0.12)**	0.46 (0.12)**	0.62 (0.12)**	0.51 (0.12)**	0.64
Potassium (mEq/L)	0.04 (0.02)	0.04 (0.02)	0.05 (0.02)*	0.09 (0.02)**	0.38
Body weight (kg)	-1.23 (0.07)**	-1.40 (0.07)**	-1.39 (0.07)**	-1.48 (0.07)**	0.08
Systolic blood pressure (mmHg)	-1.7 (0.8)*	-4.5 (0.8)**	-4.2 (0.8)**	-4.0 (0.8)**	0.06
Diastolic blood pressure (mmHg)	-1.0 (0.6)	-1.8 (0.6)**	-1.3 (0.6)*	-1.8 (0.6)**	0.74
$eGFR_{MDRD}$[a] (ml/min/1.73m^2)	-2.4 (0.5)**	-3.2 (0.5)**	-4.5 (0.5)**	-5.0 (0.5)**	0.001
$eGFR_{CKD\text{-}EPI}$[a] (ml/min/1.73m^2)	-0.6 (0.3)*	-1.4 (0.3)***	-1.9 (0.3)***	-2.2 (0.3)***	0.0004
Creatinine (mg/dL)	0.025 (0.005)**	0.032 (0.004)**	0.044 (0.005)**	0.047 (0.004)**	0.001

At week 52	Quartile 1	Quartile 2	Quartile 3	Quartile 4	P
N	193	194	194	194	
HbA1c (mmol/mol)	-9.02 (0.48)**	-8.28 (0.48)**	-9.33 (0.47)**	-7.97 (0.48)**	0.14
HbA1c (%)	-0.83 (0.04)**	-0.76 (0.04)**	-0.85 (0.04)**	-0.73 (0.04)**	0.14
Fasting plasma glucose (mmol/L)	-1.80 (0.07)**	-1.81 (0.07)**	-1.71 (0.07)**	-1.66 (0.07)**	0.46
Fasting plasma glucose (mg/dL)	-32.4 (1.3)**	-32.5 (1.3)**	-30.8 (1.3)**	-30.0 (1.3)**	0.46
Sodium (mEq/L)	0.41 (0.13)**	0.42 (0.13)**	0.42 (0.12)**	0.62 (0.13)**	0.57
Potassium (mEq/L)	-0.06 (0.02)**	-0.08 (0.02)***	-0.03 (0.02)	-0.03 (0.02)	0.18
Body weight (kg)	-3.07 (0.20)**	-3.14 (0.20)**	-2.90 (0.19)**	-3.31 (0.20)**	0.49

Systolic blood pressure (mmHg)	-4.0 (0.9)**	-5.3 (0.9)**	-5.1 (0.9)**	-4.6 (0.9)**	0.77
Diastolic blood pressure (mmHg)	-2.3 (0.6)**	-3.0 (0.6)**	-2.2 (0.6)**	-2.1 (0.6)**	0.74
BNP[b] (pg/mL)	-1.00 (1.15)	-1.35 (1.15)	-2.69 (1.12)*	-1.21 (1.17)	0.70
ACR[c] (ln-transformed mg/gCr)	-0.03 (0.05)	-0.07 (0.05)	-0.12 (0.05)*	-0.15 (0.05)**	0.27
$eGFR_{MDRD}$[a] (ml/min/1.73m^2)	3.6 (0.8)**	3.5 (0.8)**	2.1 (0.8)**	1.5 (0.8)	0.13
$eGFR_{CKD\text{-}EPI}$[a] (ml/min/1.73m^2)	1.5 (0.3)***	1.0 (0.3)***	0.6 (0.3)	0.5 (0.3)	0.10
$eGFR_{CRE+CYS}$[a] (ml/min/1.73m^2)	-0.7 (0.5)	-1.7 (0.5)***	-2.3 (0.5)***	-2.8 (0.5)***	0.01
Cystatin C (mg/L)	0.03 (0.01)***	0.05 (0.01)***	0.05 (0.01)***	0.05 (0.01)***	0.04
Creatinine (mg/dL)	-0.024 (0.005)**	-0.018 (0.005)**	-0.005 (0.005)	-0.003 (0.005)	0.01

Least square mean (standard error)
a estimated glomerular filtration rate
b brain natriuretic peptide
c urine albumin-to-creatinine ratio
Analysis of covariance (ANCOVA) between the groups
Adjusted by their baseline values, age, sex and eGFR as covariates
One sample t-test vs baseline * p<0.05, ** p<0.01, *** p<0.001 vs baseline

Supplemental Table 3. Baseline predictors for the change in serum creatinine levels at week 4 and week 52

Change in creatinine at week 4		
Factors	Regression coefficient	P
Tofogliflozin 40 mg (vs. 20 mg)	0.01	0.0317
ACR = >30 mg/g Cre (vs. ACR <30 mg/g Cre)	0.01	0.0192
Estimated daily salt intake (higher 1g/day)	0.004	<0.001
BNP (higher 1 pg/mL)	0.0004	0.0019
Use of RAS inhibition drugs (yes)	0.01	0.0162

Change in creatinine at week 52		
Factors	Regression coefficient	P
HbA1c (higher 1%)	0.01	<0.001
Estimated daily salt intake (higher 1 g/day)	0.004	0.0004
BNP (higher 1 pg/mL)	0.0003	0.0388
Use of RAS inhibition drugs (yes)	0.01	0.0062

Factors remained through stepwise variable selection with p <0.05

Potential baseline predictors were dosage of tofogliflozin, use of RAS inhibition drugs (ARB and/or ACEI), age, sex, duration of diabetes, HbA1c, DBP, BNP, BMI, uric acid, creatinine, DSI levels, and ACR <30 mg/g Cre (vs. ACR = >30 mg/g Cre).

Supplemental Table 4. Predictors for change in $eGFR_{MDRD}$ from week 4 to week 52

Change in $eGFR_{MDRD}$ from week 4 to week 52		
Factors	Regression coefficient	P
Duration of diabetes (higher 1 years)	0.18	0.0042
Age (higher 1 years)	-0.12	0.0009
Men	-2.06	0.0067
HbA1c at week 4 (higher 1%)	-1.00	0.0253

Factors remained through stepwise variable selection with p <0.05

Potential baseline predictors were dosage of tofogliflozin, use of RAS inhibition drugs (ARB and/or ACEI), age, sex, duration of diabetes, variables at week 4 (HbA1c, DBP, BMI, uric acid and $eGFR_{MDRD}$), DSI levels, and ACR <30 mg/g Cre (vs. ACR = >30 mg/g Cre)

Supplemental Table 5. Predictors of change in $eGFR_{MDRD}$ from week 52 to week 54

Change in $eGFR_{MDRD}$ from week 52 to 54		
Factors	Regression coefficient	P
HbA1c at week 52 (higher 1%)	1.20	0.0155
Uric acid at week 52 (higher 1 mg/dL)	0.62	0.0380
$eGFR_{MDRD}$ at week 52 (higher 1 ml/min/1.73m^2)	-0.11	<0.001
Duration of diabetes (higher 1 year)	-0.16	0.0044

Factors remained through stepwise variable selection with p <0.05

Potential baseline predictors were dosage of tofogliflozin, use of RAS inhibition drugs (ARB and/or ACEI), age, sex, duration of diabetes, variables at week 52 (HbA1c, DBP, BNP, BMI, uric acid, $eGFR_{MDRD}$), DSI levels, and ACR <30 mg/g Cre (vs. ACR = >30 mg/g Cre).

Supplemental Table 6. Correlations between the change in $eGFR_{MDRD}$ at week 4 and week 52 and baseline continuous variables

	Change in $eGFR_{MDRD}$ at week 4			Change in $eGFR_{MDRD}$ at week 52		
Factors	n	R	P	N	R	P
Age (years)	774	0.02	0.5816	686	-0.08	0.0462
Body weight (kg)	774	-0.04	0.2261	686	-0.05	0.1846
Body mass index (kg/m^2)	774	-0.07	0.0560	686	-0.06	0.1251
HbA1c (%)	774	-0.11	0.0017	686	-0.15	<0.001
Fasting plasma glucose (mmol/L)	774	-0.13	0.0003	686	-0.12	0.0015
Sodium (meq/L)	774	0.05	0.1308	686	0.05	0.2123
Potassium (meq/L)	774	0.03	0.3556	686	0.02	0.6630
Systolic blood pressure (mmHg)	774	-0.10	0.0061	686	-0.04	0.2560
Diastolic blood pressure (mmHg)	774	-0.06	0.0950	686	-0.03	0.3788
Duration of diabetes mellitus (years)	774	-0.06	0.1057	686	0.02	0.5319
Creatinine (mg/dL)	774	0.20	<0.001	686	-0.01	0.7716
Cystatin C (mg/L)	774	0.07	0.0638	686	-0.09	0.0183
$eGFR_{MDRD}$[a] ($ml/min/1.73m^2$)	774	-0.23	<0.001	686	-0.02	0.6843
Uric acid (mg/dL)	774	0.06	0.0919	686	0.02	0.5304
BNP[b] (pg/mL)	774	-0.08	0.0228	686	-0.08	0.0494
Urinary Na excretion (mmol/day)	774	-0.22	<0.001	686	-0.09	0.0165
Urinary K excretion (mmol/day)	774	-0.16	<0.001	686	-0.02	0.5454
Estimated daily salt intake (g/day)	774	-0.22	<0.001	686	-0.09	0.0165

a estimated glomerular filtration rate

b brain natriuretic peptide

R, Pearson's product-moment correlation coefficient.

Supplemental Table 7. Change in $eGFR_{MDRD}$ at week 4 and week 52 according to baseline categorical variables

Factors		Change in $eGFR_{MDRD}$ at week 4 n	Mean (SD)		n	Mean (SD)	p
Sex	Men	512	-3.4 (7.4)	Women	262	-4.2 (6.8)	0.1586
Tofogliflozin	20mg	235	-2.8 (7.1)	40mg	539	-4.1 (7.2)	0.0235
Concomitant antihypertensive drugs	yes	366	-4.2 (6.8)	No	408	-3.2 (7.6)	0.0665
ARB	yes	275	-4.5 (6.9)	No	499	-3.2 (7.3)	0.0164
ACEI[a]	yes	18	-1.9 (5.3)	No	756	-3.7 (7.3)	0.2943
ARB and/or ACEI	yes	291	-4.4 (6.8)	No	484	-3.2 (7.4)	0.0368
CCB[b]	yes	218	-4.4 (7.0)	No	556	-3.4 (7.3)	0.0946
Beta-blockers	yes	27	-3.8 (6.9)	No	747	-3.7 (7.2)	0.9154
Diuretics	yes	64	-4.1 (7.3)	No	710	-3.6 (7.2)	0.6480
ACR[c] category	< 30	519	-3.2 (7.1)	= > 30	255	-4.6 (7.4)	0.0085

Factors		The change in $eGFR_{MDRD}$ at week 52 n	Mean (SD)		n	Mean (SD)	p
Sex	Men	451	2.0 (8.8)	Women	235	3.3 (11.6)	0.1159
Tofogliflozin	20mg	203	2.6 (10.8)	40mg	483	2.4 (9.4)	0.7247
Concomitant antihypertensive drugs	yes	327	1.4 (9.4)	No	359	3.4 (10.2)	0.0096
ARB	yes	245	0.6 (8.8)	No	441	3.5 (10.3)	0.0002
ACEI[a]	yes	16	3.9 (8.5)	No	670	2.4 (9.9)	0.5538
ARB and/or ACEI	yes	260	0.8 (8.8)	No	426	3.4 (10.3)	0.0007
CCB[b]	yes	198	1.6 (9.1)	No	488	2.8 (10.2)	0.1339
Beta-blockers	yes	23	2.2 (15.8)	No	663	2.4 (9.6)	0.9138
Diuretics	yes	56	1.2 (9.3)	No	630	2.5 (9.9)	0.3324
ACR[c] category	< 30	459	2.9 (9.7)	= > 30	227	1.4 (10.2)	0.0629

a ACE inhibitor

b calcium channel blocker

c urine albumin-to-creatinine ratio

Analyses were performed by ANOVA.
